# Outcomes of targeted treatment in immunocompromised patients with asymptomatic or mild COVID-19: a retrospective study

**DOI:** 10.1038/s41598-023-42727-5

**Published:** 2023-09-16

**Authors:** M. Lahouati, C. Cazanave, A. Labadie, P. Gohier, L. Guirlé, A. Desclaux, M. Gigan, D. Malvy, S. Pedeboscq, F. Xuereb, A. Duvignaud, The Bordeaux COVID-19 Treatment Group, Laure Barthod, Pantxika Bellecave, Jean-Frédéric Blanc, Elodie Blanchard, Fabrice Bonnet, Fabrice Camou, Mathilde Carrer, Charles Cazanave, Faiza Chermak, Lionel Couzi, Amaury Daste, Frédéric-Antoine Dauchy, Victor De Ledinghen, Charlotte Domblides, Pierre Duffau, Hervé Dutronc, Alexandre Duvignaud, Maxime Faure, Edouard Forcade, Nahéma Issa, Hannah Kaminski, Jean-Baptise Hiriart, Marin Lahouati, Julie Leitao, Maëlig Lescure, Estibaliz Lazaro, Isabelle Maachi, Didier Neau, Duc Nguyen, Karine Nubret, Stéphane Pédeboscq, Thierry Pistone, Frédérique Pribat, Mathilde Puges, Aurélie Ruet, Camille Tumiotto, Marie-Anne Vandenhende, Gaétane Wirth

**Affiliations:** 1 Service de Pharmacie Clinique, Hôpital Pellegrin, Centre Hospitalo-Universitaire de Bordeaux, Place Amélie Raba Léon, 33076 Bordeaux, France; 2 Inserm, UMR 1034, Biology of Cardiovascular Diseases, Université de Bordeaux, Pessac, France; 3 Service des maladies infectieuses et tropicales, CHU de Bordeaux, 33076 Bordeaux, France; 4 Inserm UMR 1219, IRD EMR 271, Bordeaux Population Health, Université de Bordeaux, 33076 Bordeaux, France; 5 Service de virologie, CHU de Bordeaux, 33076 Bordeaux, France; 6 Service d’oncologie digestive, CHU de Bordeaux, 33076 Bordeaux, France; 7 Service de pneumologie, CHU de Bordeaux, 33076 Bordeaux, France; 8 Service de Medecine interne et maladies infectieuses, CHU de Bordeaux, 33076 Bordeaux, France; 9 Service reanimation medicale, CHU de Bordeaux, 33076 Bordeaux, France; 10 Service d’hépato-gastro-enterologie, CHU de Bordeaux, 33076 Bordeaux, France; 11 Service de nephrologie, CHU de Bordeaux, 33076 Bordeaux, France; 12 Service d’oncologie medicale, CHU de Bordeaux, 33076 Bordeaux, France; 13 Service d’immunologie clinique, CHU de Bordeaux, 33076 Bordeaux, France; 14 Service de cardiologie, CHU de Bordeaux, 33076 Bordeaux, France; 15 Service d’hématologie, CHU de Bordeaux, 33076 Bordeaux, France; 16 Service de Médecine interne, CHU de Bordeaux, 33076 Bordeaux, France; 17 Service de neurologie, CHU de Bordeaux, 33076 Bordeaux, France

**Keywords:** Infectious diseases, Viral infection

## Abstract

The aim of this study was to describe the outcomes of targeted COVID-19 treatments in immunocompromised patients with asymptomatic or mild COVID-19 during the period of expansion of the different Omicron subvariants in France. A retrospective monocentric observational study was performed. All immunocompromised patients aged 18 or more, with asymptomatic SARS-CoV-2 infection or mild COVID-19, and who had received a targeted treatment with sotrovimab, tixagevimab/cilgavimab, nirmatrelvir/ritonavir or remdesivir at the Bordeaux University Hospital from 1st January 2022 to 31st December 2022 were eligible. The primary outcomes of interest was defined as a composite of either (i) progression to moderate (WHO-Clinical Progression Scale at 4 or 5) or severe COVID-19 (WHO-CPS ≥ 6), or (ii) the occurrence of COVID-19-related death. The secondary outcomes of interest were the components of the primary outcome. Outcomes were collected until day 30 after targeted treatment administration or at discharge for patients still hospitalised in relation with COVID-19 at day 30. 223 immunocompromised patients received targeted treatment for asymptomatic SARS-CoV-2 infection or mild COVID-19: 114 received sotrovimab, 50 tixagevimab/cilgavimab, 49 nirmatrelvir/ritonavir, and 10 remdesivir. Among 223 treated patients, 10 (4.5%) progressed to moderate or severe disease: three patients (1.3%) progressed to moderate COVID-19 and 7 (3.1%) patients progressed to severe disease. Among them, 4 (1.8%) died of COVID-19. More than 95% of immunocompromised patients with asymptomatic SARS-CoV-2 infection or mild COVID-19 treated by targeted therapies during the Omicron subvariants era did not progress to moderate or severe disease.

## Introduction

Since more than three years, COVID-19 led to 6.9 million deaths worldwide^1^. Omicron became the major circulating SARS-CoV-2 variant in France since December 2021^2^. Although the disease due to Omicron is less severe than with the historical strain in the general population^3^, several studies showed that immunocompromised patients still have a significant risk of bad outcome^4,5^. Moreover, Omicron subvariants (BA.1, BA.2, BA.4/5 and BQ.1.1) that were circulating in 2022 were associated to a reduction in vaccine in vitro neutralization activity^6,7^.

Several targeted therapies are available for the early treatment of COVID-19 in at risk populations in France: the monoclonal antibodies (mAb) sotrovimab and tixagevimab/cilgavimab, and the small antiviral compounds nirmatrelvir (3 M Protease inhibitor) and remdesivir (nucleoside analogue). These treatments have demonstrated their efficacy to prevent the progression to severe disease when given early to at-risk patients^8–11^. However immunocompromised patients were poorly or not at all represented in efficacy trials, therefore making it hard to conclude on the real benefit of these treatments in this at-risk population^12^. Moreover, most of these trials have been conducted before the Omicron era. Of note, targeted treatments have variable activity on the different Omicron subvariants^6,7,13,14^. Thus, there is a knowledge gap regarding the efficiency of targeted treatments in immunocompromised patients with COVID-19 in the Omicron era.

The aim of this study was to describe outcomes of targeted COVID-19 treatments in immunocompromised patients with asymptomatic or mild COVID-19 cared at Bordeaux University Hospital during the period of expansion of the different Omicron subvariants in France.

## Methods

### Study population

A retrospective monocentric observational study was performed. All immunocompromised patients aged 18 or more, with asymptomatic SARS-CoV-2 infection or mild COVID-19 who were considered as high to very high risk of progressing to severe disease according to French guidelines^15^, and who had received a targeted treatment with sotrovimab, tixagevimab/cilgavimab, nirmatrelvir/ritonavir or remdesivir at Bordeaux University Hospital from 1st January 2022 to 31st December 2022 were eligible.

SARS-CoV-2 infection was defined as having a positive SARS-CoV-2 diagnostic test (either nasopharyngeal RT-PCR or antigenic test).

Patients hospitalised for COVID-19 or with severe COVID-19 at treatment initiation were excluded. Disease severity was assessed according to the WHO clinical progression scale (WHO-CPS), (Annex 1).

### Omicron subvariants circulation

In France, data on circulating variant and subvariant were collected by France national surveillance resaux (SiDEP). Omicron became the major circulating variant in late December 2021. However, the predominant Omicron subvariant (i.e. representing > 50% of screening tests) changed over time during the study period: the main circulating subvariant was BA.1 between January 2022 and February 2022; BA.2 between March 2022 and June 2022, BA.4/BA.5 between June 2022 and November 2022. At last, BQ.1.1, was the main circulating subvariant in France since mid- November 2022^2^.

In this study, it was not possible to characterize the subvariant responsible for each individual infection from routine RT-PCR results because subvariant screening was not performed for all positive tests in France.

### Data collection and definitions

Demographic data, medical history, comorbidities, vaccination status, previous pre-exposure prophylaxis with mAb, causes of immunosuppression, targeted treatments received, and treatment outcomes were collected from electronic health records. Patients were contacted by phone if follow-up information was not directly available.

The main causes of immunosuppression were classified as follows: solid organ transplantation (SOT), anti-CD20 therapy with last infusion received less than 12 months ago, active haematological or solid malignancy currently treated with chemotherapy other than anti-CD20 therapy, and allogenic stem cell transplantation. Other causes of immunosuppression were also collected.

Patients were considered as fully vaccinated if they had received either at least 3 doses of a mRNA-based vaccine or if they had received two doses of a mRNA-based vaccine and had a documented SARS-CoV-2 infection^16^. Seropositive status was defined as having an anti-spike IgG titer over 7 binding antibody units (BAU)/mL (detection threshold), evaluated at least 15 days after the 3rd dose of a mRNA-based vaccine or after a documented natural infection. Serological status was considered as unknown if data were either missing, or uninterpretable, or if a patient had received pre-exposure prophylaxis with monoclonal antibodies (casirivimab/imdevimab or tixagevimab/cilgavimab) within 6 months before testing.

### Targeted treatments

Sotrovimab is available since 21st January 2022 at our centre. The dose regimen used was 500 mg administered intravenously.

Nirmatrelvir/ritonavir is available in our centre since 4th February 2022. The dose regimen used was 300 mg of nirmatrelvir and 100 mg of ritonavir administered orally twice daily for 5 days. In patients with moderate renal impairment (eGFR ≥ 30 to < 60 mL/min), the dose of nirmatrelvir was halved (150 mg per intake) while the dose of ritonavir remained unchanged. Nirmatrelvir/ritonavir was not used in patients with severe renal impairment (eGFR < 30 mL/min).

The tixagevimab/cilgavimab association was available in compassionate use for the treatment of COVID-19 since 14th January 2022 in France. The dose regimen used for curative treatment was 300 mg of tixagevimab and 300 mg of cilgavimab administered intravenously.

Remdesivir is available since 2020 at our centre. The dose used was 200 mg once a day at day 1, then 100 mg once a day on day 2 and day 3, given by intravenous infusion.

None of patient was treated by molnupiravir due to its unavailability in France.

The choice of the treatment was made by the treating physician, advised by infectious disease physicians and clinical pharmacists. It depended on the knowledge of the main circulating Omicron subvariants in France at that time, the availability of the different targeted treatments at our center, and on patient's contraindications, notably drug-drug interactions for nirmatrelvir/ritonavir.

French guidelines recommended to start the treatment as soon as possible, ideally within five days, after symptoms onset/positive testing^15^. For some patients, the treatment was started later because they were considered at high risk of progressing to severe disease and the risk/benefit balance of a late treatment was deemed favourable by the treating physicians.

### Outcomes

The primary outcomes of interest was defined as a composite of either (i) progression to moderate (WHO-Clinical Progression Scale at 4 or 5) or severe COVID-19 (WHO-CPS ≥ 6), or (ii) the occurrence of COVID-19-related death. The secondary outcomes of interest were the components of the primary outcome. Outcomes were collected until day 30 after targeted treatment administration or at discharge for patients still hospitalised in relation with COVID-19 at day 30.

### Statistical analysis

Continuous variables were presented as medians and interquartile range (IQR). Categorical variables were presented as percentage. Univariate analysis was performed using Fischer’s exact test qualitative variables and ANOVA or Kruskal–Wallis tests for continuous variables. A p-value < 0.05 was considered as significant (two-sided).

### Compliance with ethics guidelines

Our study was conducted following the French MR-004 reference methodology regarding the secondary use and processing of already collected personal health data for the purpose of clinical research studies and evaluation of medical practices. The MR-004 relies on prior patients’ information and opposition right. It also obliges the data controller to appoint a Data Protection Officer (DPO). Hence, eligible patients were informed prior to the collection of their personal health data for secondary research use. None of them objected. This study complies with the GDPR (EU General Data Protection Regulation) and was approved by the Bordeaux University Hospital’s DPO and by the National Data Protection and Privacy Commission (CNIL) under the reference CHUBX2022RE0304.

## Results

### Patients’ characteristics

223 immunocompromised patients infected with SARS-CoV-2, of whom 187 (84%) had mild symptoms, received a targeted treatment during the study period. The median age was 59 years (IQR 42–68), 124 (56%) were male, and the median BMI was 24.8 kg/m^2^ (IQR 21–28). The three main causes of immunosuppression were SOT (n = 81; 36%), anti-CD20 therapy (n = 59; 27%), and chemotherapy for haematological malignancy without anti-CD20 agent (n = 32; 14%). 16 (7%) patients had received allogenic stem cell transplantation. The median delay between allogenic stem cell transplantation and SARS-CoV-2 infection was 252 days (IQR 137–1095; range 1–5345). Most patients were fully vaccinated (n = 167; 75%). 100 (45%) patients had a serological evaluation within 3 months before infection. Overall, 35/100 patients had a negative SARS-Cov2 serological status.

Only 29 (13%) patients had received pre-exposure prophylaxis with monoclonal antibodies within 6 months prior to infection, of whom 26 had received an infusion of tixagevimab/cilgavimab. The median delay between pre-exposure prophylaxis and infection was 80 days (IQR 59–148; range 22–184). Patients’ characteristics are reported in Table 1.

**Table 1:** Patients’ characteristics (N = 223).

	Total N = 223	Sotrovimab N = 114	Tixagevimab/ cilgavimab N = 50	Nirmatrelvir/ritonavir N = 49	Remdesivir N = 10	*p*-value
Age (years); median (IQR)	59 (42; 68)	55 (40.25; 67.75)	60 (49; 66)	66.5 (47.5; 73.5)	63 (45.2; 67.5)	0.15
Sex						
M; n (%)	124 (56)	64 (56)	24 (48)	28 (57)	8 (80)	0.31
F; n (%)	99 (44)	50 (44)	26 (52)	21 (43)	2 (20)	–
BMI, median (IQR)	24.8 (21.5; 28)	24.9 (21.6; 29.6)	24.7 (21.4; 26.7)	24.3 (22.8; 27.7)	25.3 (22.3; 28;7)	0.62
Comorbidities						
BMI > 30 kg/m^2^; n (%)	38 (17)	24 (21)	6 (12)	6 (12)	2 (20)	0.37
Diabetes mellitus; n (%)	44 (20)	18 (16)	15 (30)	7 (14)	4 (40)	**0.04**
Chronic heart failure; n (%)	15 (7)	8 (7)	4 (8)	3 (6)	0	0.97
Chronic respiratory failure; n (%)	8 (4)	3 (3)	1 (2)	4 (8)	0	0.35
GFR < 30 ml/min/1.73 m^2^; n (%)	21 (9)	10 (9)	7 (14)	2 (4)	2 (20)	0.2
End-stage kidney disease with dialysis; n (%)	4 (2)	2 (2)	2 (4)	0 (0)	0	0.6
Causes of immunosuppression						
Solid organ transplantation; n (%)	81^a^ (36)	41^a^ (35)	34^a^ (68)	0 (0)	6 (60)	** < 0.001**
Kidney; n (%)	25^a^ (11)	19^a^ (17)	5^a^ (10)	0 (0)	1 (10)	–
Heart; n (%)	21^a^ (9)	12^a^ (11)	4 (8)	0 (0)	5 (50)	–
Liver; n (%)	14 (6)	5 (4)	9 (18)	0 (0)	0 (0)	–
Lung; n (%)	17 (8)	4 (4)	13 (26)	0 (0)	0 (0)	–
Multiorgan; n (%)	4 (2)	1 (1)	3 (6)	0 (0)	0 (0)	–
Anti-CD20 therapy; n (%)	59^a^ (27)	32^a^ (28)	9 (18)	16 (32)	2 (20)	0.38
Anti-CD20 based chemotherapy for haematological malignancy; n (%)	25^a^ (11)	9^a^ (8)	3 (6)	11 (22)	2 (20)	**0.02**
Anti-CD20 for multiple sclerosis; n (%)	20 (9)	17 (15)	1 (2)	2 (4)	0 (0)	**0.02**
Anti-CD20 for autoimmune disease^b^; n (%)	11 (5)	5 (4)	3 (6)	3 (6)	0 (0)	0.86
Anti-CD20 for solid organ transplantation; n (%)	3^a^ (1)	1^a^ (1)	2^a^ (2)	0	0 (0)	0.36
Chemotherapy for haematological malignancy without anti-CD20 therapy; n (%)	32 (14)	18 (16)	4 (8)	9 (18)	1 (10)	0.46
Chemotherapy for solid cancer; n (%)	19 (9)	9 (8)	1 (2)	9 (18)	0 (0)	**0.03**
Allogenic stem cell transplantation; n (%)	16 (7)	5 (4)	3 (6)	7 (14)	1 (10)	0.12
Other; n (%)	20 (9)	11 (10)	1 (2)	8 (16)	0 (0)	0.06
Vaccination status						
Fully vaccinated; n (%)	167 (75)	91 (79)	39 (78)	30 (61)	8 (80)	0.17
Not fully vaccinated; n (%)	12 (5)	7 (6)	0 (0)	5 (10)	0 (0)	–
Unvaccinated; n (%)	6 (3)	3 (3)	0 (0)	3 (6)	0 (0)	–
Vaccination status unknown; n (%)	37 (16)	13 (11)	11 (22)	11 (22)	2 (20)	–
Seropositive; n (%)	86 (36)	45 (39)	26 (52)	14 (29)	1 (10)	** < 0.001**
Seronegative; n (%)	35 (16)	27 (24)	5 (10)	0 (0)	2 (20)	–
Serologic status unknown; n (%)	73 (32)	34 (30)	14 (28)	23 (47)	2 (20)	–
Serologic status uninterpretable; n (%)	29 (13)	8 (7)	5 (10)	12 (24)	4 (40)	–
IgG anti-spike (BAU/mL); median (IQR)	156 (0; 726)	49.5 (0; 324.75)	188 (75.5; 1067.5)	1162 (381; 2875)	0 (0; 136)	** < 0.001**
IgG anti-spike < 264 BAU/mL; n (%)	76 (34)	52 (46)	19 (38)	3 (6)	2 (20)	** < 0.001**
Pre-exposure prophylaxis with monoclonal antibodies within 6 months						
Yes; n (%)	29 (13)	8 (7)	5 (10)	12 (24)	4 (40)	** < 0.001**
Tixagevimab/cilgavimab; n (%)	26 (12)	5 (3)	5 (10)	12 (24)	4 (40)	–
Casirivimab/imdevimab; n (%)	3 (1)	3 (3)	0 (0)	0 (0)	0 (0)	–
No; n (%)	194 (87)	106 (93)	45 (90)	37 (76)	6 (60)	–
Variant						
Omicron; n (%)	140 (63)	79 (69)	36 (72)	18 (37)	7 (70)	** < 0.001**
Non-Omicron; n (%)	2 (1)	0	0	0	2 (10)	–
Unknown; n (%)	82 (36)	35 (31)	14 (28)	31 (63)	1 (10)	–
Clinical status						
Symptomatic	187 (84)	98 (86)	41 (82)	38 (78)	10 (100)	0.31
Asymptomatic	36 (15)	16 (14)	9 (18)	11 (22)	0 (0)	–

^a^Two SOT recipients in the sotrovimab group and two in the tixagevimab/cilgavimab group were also receiving an anti-CD20 therapy.

^b^Wegener's granulomatosis (n = 1), auto-immune cerebellitis (n = 1), neuromyelitis optica (n = 2), chronic polyradiculoneuropathy (n = 1), Lewis-Sumner syndrome (n = 1), ANCA-associated vasculitis (n = 1); glomerulonephritis (n = 1), microscopic polyangiitis (n = 1), idiopathic thrombocytopenic purpura (n = 1) and systemic lupus erythematosus (n = 1).

Significant values are in bold.

### Targeted treatments

**Figure 1:**
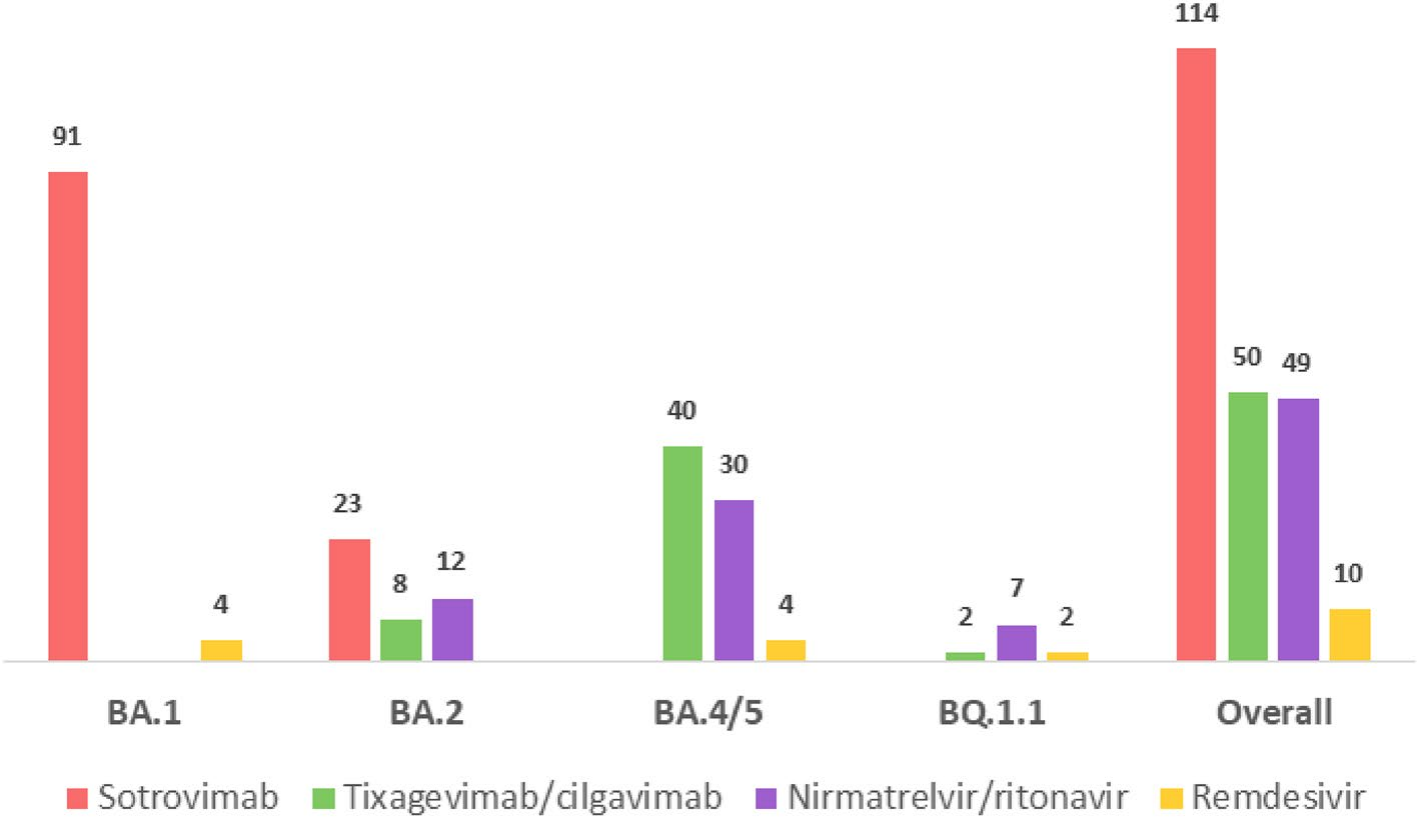
Targeted treatments administrations according to the predominant circulating Omicron subvariants (N = 223).

114 (51%) immunocompromised patients were treated with sotrovimab, 50 (22%) with tixagevimab/cilgavimab, 49 (22%) with nirmatrelvir/ritonavir and 10 (4%) with remdesivir. The median delay between symptoms onset (or diagnostic test in asymptomatic patients) and treatment administration was 2 days (IQR 1–4; range 0–10). Targeted treatments received according to the predominant circulating Omicron subvariants in France at that time are detailed in Fig. 1. No adverse event was reported and no treatment was discontinued.

### Outcomes

Among 223 treated patients, 10 (4.5%) progressed to moderate or severe COVID-19. Outcomes according to targeted treatment are reported in Table 2.

**Table 2:** Outcomes of interest by day 30 or at patient discharge.

Outcomes	Description	Sotrovimab N = 114	Tixagevimab/cilgavimab N = 50	Nirmatrelvir/ritonavir N = 49	Remdesivir N = 10	Total N = 223
Moderate disease	Hospitalised: no oxygen therapy (WHO-CPS 4) (n; %)	0 (0)	0 (0)	1 (3)	0 (0)	1 (0.5)
	Hospitalised: low-flow oxygen by mask or nasal prongs (WHO-CPS 5) (n; %)	1 (1)	0 (0)	1 (2)	0 (0)	2 (0.8)
Severe disease	Intensive care unit for COVID-19 and survived^a^ (WHO-CPS 6 to 9) (n; %)	1 (1)	0 (0)	1 (3)	1 (20)	3 (1.3)
	COVID-19-related death^b^ (WHO-CPS 10) (n; %)	3 (3)	1 (2)	0 (0)	0 (0)	4 (1.8)

^a^One patient received high-flow oxygen supplementation and two patients received NIV.

^b^One patient received NIV, one received invasive mechanical ventilation and two patients were not admitted to intensive care unit due to comorbidities.

Three patients (1. 3%) progressed to moderate COVID-19: one patient (0.4%) was hospitalised without oxygen supplementation and two (0.9%) were hospitalised and required low flow oxygen supplementation without needing intensive care unit.

Seven patients (3.1%) progressed to severe COVID-19: 3 (1.3%) were admitted to intensive care unit for COVID-19 and survived and 4 (1.8%) died as a consequence of COVID-19.

Overall, 8 (3.6%) patients died of any causes.

All patients who progressed to pneumonia requiring oxygen supplementation (WHO-CPS score ≥ 5) were treated by dexamethasone for 10 days. One patient received tocilizumab. Median duration of oxygen supplementation was 7 days (IQR 2.75–9; range 2–17). Five patients were hospitalised in intensive care unit (4 received non-invasive ventilation and 1 required intubation and mechanical ventilation). Among them, 2 died (21 and 75 days after treatment administration). The median duration of hospitalization in intensive care unit was 4 days (IQR 3–19; range 2–40). Two patients were not admitted to intensive care unit, due to comorbidities, and died because of COVID-19.

The individual characteristics of patients who presented outcomes of interests are reported in supplementary Table S1.

## Discussion

This study describes outcomes of targeted COVID-19 treatments in 223 immunocompromised patients during periods with various circulating Omicron subvariants in France (BA.1, BA.2, BA.4/5 and BQ.1.1). Overall, 4.5% progressed to moderate or severe disease and 1.8% died of COVID-19.

Main causes of immunosuppression in our cohort were SOT recipients (36%), anti-CD20 therapy (27%) and chemotherapy for haematological malignancy without anti-CD20 therapy (14%). Despite disparate COVID-19 related outcomes in immunocompromised patients, these immunocompromising conditions lead to a high risk of COVID-19 progression^17,18^. Chavarot et al*.*^19^ reported severe outcomes in 35% of SOT recipients with Omicron infection which had not received sotrovimab. Thus, preventive (vaccines, pre-exposure prophylaxis) and curative (targeted treatment) strategies are essential for this vulnerable population.

In immunocompromised patient who received a 3 doses mRNA-based vaccine, vaccine effectiveness against COVID-19 related hospitalisation was 88% in the Delta era^20^. In our study, a high rate of patients (75%) were fully vaccinated. However, with Omicron variant evolution, humoral immune response induced by vaccines became less effective^6^.

In this study, few patients (n = 35; 15%) had received pre-exposure prophylaxis, a strategy that reduces the risk of COVID-19 related hospitalization in immunocompromised people^21,22^. Of note, among the 29 patients who had received pre-exposure prophylaxis within 6 months prior to infection, only 1 experienced an unfavourable outcome. Further studies should be conducted to further evaluate the efficacy of targeted curative treatments in immunocompromised patients who had previously received an efficient pre-exposure prophylaxis strategy.

In our sample, 4.4% (5/114) and 2.6% (3/114) of patient treated with sotrovimab progressed to moderate or severe COVID-19 and died respectively. This is consistent with other studies^21,23–26^. Of note, 20.2% (23/114) of these patients were treated during the BA.2 circulation period, a subvariant on which the neutralizing activity of sotrovimab is significantly reduced compared to BA.1. Over this period, 8.7% (2/23) of patients receiving sotrovimab progressed to moderate or severe disease, and 4.3% (1/23) died of COVID-19. Due to the small sample size, the unavailability of individual subvariant screening, and the absence of control strategy, it is not possible to formally compare these two periods. However, we cannot rule out that less favourable outcomes during the BA.2 period compared to the BA.1 period were related to a lesser activity of sotrovimab on BA.2. Finally, sotrovimab retains some activity on BQ.1.1 and may be an alternative for patients infected with this Omicron subvariant who have contra-indication to other curative treatment strategies^7^.

Immunocompromised patients treated by tixagevimab/cilgavimab are poorly described in the literature^8,21,23^. In this study, only 2% (1/50) progressed to severe disease and died during BA.4/5 period. To our knowledge, this is one of the largest published cohort of immunocompromised patients treated by tixagevimab/cilgavimab.

There are limited evidence on outcomes of immunocompromised patient treated by nirmatrelvir/ritonavir for mild COVID-19^11,21,27–30^. Minoia et al. reported a higher rate (10.9%) of progression to COVID-19 requiring oxygen supplementation among immunocompromised patient treated by nirmatrelvir/ritonavir than in our study (4%). Compared to Minoia cohort, we included several type of immunocompromised condition, while they focused on patient with active haematological malignancy. Makuska et al*.* reported that 5.8% of patient with haematological malignancy treated by small antivirals (nirmatrelvir/ritonavir or remdesivir) progressed to severe COVID-19 requiring oxygen supplementation, however, about 20% of their cohort did not have an active haematological malignancy. Nirmatrelvir/ritonavir prescription should be done with caution in some patients, particularly in SOT recipients, due to drug-drug interactions between ritonavir and calcineurin or mammalian target of rapamycine inhibitors^31^. In our cohort, no SOT recipients received nirmatrelvir/ritonavir, reflecting the high reluctance of treating physicians to use this strategy in this population. However, this drug retains its full activity on all the Omicron subvariants in circulation so far. Therefore, it is of paramount important to explore avenues that would make it easier to prescribe in situations at risk of drug-drug interaction.

Concluding on outcomes of immunocompromised patients treated by remdesivir is difficult because of the very limited sample size. Remdesivir represents an alternative to nirmatrelvir/ritonavir when the latter is contraindicated and the use of mAb is not possible due their lack of activity on some subvariants.

This study has several limitations. First, some treatment groups were small and therefore the number of unfavourable outcomes was very low. Then, our study population is not exhaustive: immunocompromised patients treated by nirmatrelvir/ritonavir in community care (drug not dispensed by our hospital’s pharmacy) were not included in this study due to our data collection method. Moreover, our study is a retrospective study, the population is heterogenous and without a control group. Thus, we cannot conclude on targeted treatment efficacy.

In addition, because of the heterogeneity of our study population, we could not match subpopulations receiving the various treatment strategies for risk factors of COVID-19 progression.

Importantly, we were unable to assess the impact of targeted treatment on viral clearance: data regarding follow-up RT-PCR were not available (mostly performed in community laboratories).

Lastly, the unavailability of routine individual subvariant characterization prevented us to make correlations between Omicron subvariants and treatment outcomes. Instead, we had to base our analysis on the predominant subvariant circulating in France at the time of treatment. Hence, the choice of the treatment strategy by treating physicians was most often based on an indirect knowledge of the circulating Omicron subvariants based on the current epidemiological situation in France, as well as on patient’s comorbidities and potential drug-drug interactions. This makes the results of the present study particularly relevant from the perspective of the clinician, as they reflect the real-life use of COVID-19 targeted treatment strategies.

In conclusion, more than 95% of immunocompromised patients with asymptomatic SARS-CoV-2 infection or mild COVID-19 treated by targeted therapies during the Omicron subvariants era did not progress to moderate or severe disease.

However, in a context where several of current available therapies and vaccines have a reduced or no activity on circulating Omicron subvariants compared to that which they had on ancestral SARS-CoV-2 strains^32^, randomized trials evaluating the efficacy of available and newly developed targeted treatment strategies in immunocompromised patients are urgently needed^12^.

### Supplementary Information

Supplementary Information 1.

Supplementary Table S1.

## Data Availability

The datasets generated during and/or analysed during the current study are available from the corresponding author on reasonable request.
