# Supplementary material for: Outcomes of targeted treatment in immunocompromised patients with asymptomatic or mild COVID-19: a retrospective study
Source: Sci Rep. 2023 Sep 16;13:15357. doi: 10.1038/s41598-023-42727-5 (PMC10505186; doi:10.1038/s41598-023-42727-5)
Supplement: Supplementary file 1 — Supplementary Information 1. [file 41598_2023_42727_MOESM1_ESM.docx]

**Annex 1: WHO clinical progression scale (WHO-CPS)**

| **WHO-CPS** | **Descriptor** | **Score** |
| --- | --- | --- |
| **Uninfected** | Uninfected; non-viral RNA detected | 0 |
| **Ambulatory mild disease** | Asymptomatic; viral RNA detected | 1 |
|  | Symptomatic; Independent | 2 |
|  | Symptomatic; Assistance needed | 3 |
| **Hospitalised : moderate disease** | Hospitalised; No oxygen therapy | 4 |
|  | Hospitalised; oxygen by mask or nasal prongs | 5 |
| **Hospitalised :**  **severe disease** | Hospitalised; oxygen by NIV or High flow | 6 |
|  | Intubation and Mechanical ventilation, pO2/FIO2>=150 OR SpO2/FIO2>=200 | 7 |
|  | Mechanical ventilation(pO2/FIO2 <150 OR SpO2/FIO2 <200 OR vasopressor (norepinephrine >0.3 microg/kg/min) | 8 |
|  | Mechanical ventilation(pO2/FIO2 <150 and vasopressor (norepinephrine >0.3 microg/kg/min) OR Dialysis OR ECMO | 9 |
| **Dead** | Dead | 10 |
