# Supplementary material for: Outcomes of targeted treatment in immunocompromised patients with asymptomatic or mild COVID-19: a retrospective study
Source: Sci Rep. 2023 Sep 16;13:15357. doi: 10.1038/s41598-023-42727-5 (PMC10505186; doi:10.1038/s41598-023-42727-5)
Supplement: Supplementary file 2 — Supplementary Table S1. [file 41598_2023_42727_MOESM2_ESM.docx]

**Supplementary table S1: Characteristics of patients who experienced an outcome of interest (N=10)**

| **Patient** | **Age (years)** | **Sex** | **Targeted treatment** | **Cause of immunosuppression** | **Vaccination (doses)** | **Pre-exposure prophylaxis** | **IgG anti-spike (BAU/mL)** | **Predominant circulating Omicron subvariant at the time of infection** | **Outcomes** |
| --- | --- | --- | --- | --- | --- | --- | --- | --- | --- |
| 1 | 66 | M | Sotrovimab | SOT (cardiac) | Unknown | No | 174 | BA.1 | Intensive care unit hospitalization and high-flow oxygen supplementation |
| 2 | 68 | M | Sotrovimab | Rituximab for neuromyelitis optica | 3 | No | < 7 | BA.1 | Covid-19-related death |
| 3 | 44 | M | Sotrovimab | SOT (kidney) | Unknown | No | 28 | BA.1 | Covid-19-related death |
| 4 | 83 | M | Sotrovimab | Untreated chronic lymphoid leukemia | 4 | No | < 7 | BA.2 | Hospitalised and required low flow oxygen supplementation |
| 5 | 84 | M | Sotrovimab | Hodgkin lymphoma treated by brentuximab-vedotin | 4 | No | Unknown | BA.2 | Covid-19-related death |
| 6 | 82 | M | Remdesivir | Chronic lymphoid leukemia treated by acalabrutinib | 3 | No | Unknown | BA.1 | Intensive care unit hospitalization and non-invasive ventilation |
| 7 | 63 | M | Nirmatrelvir/  ritonavir | Chronic lymphoid leukemia treated by rituximab and venetoclax | 3 | No | Unknown | BA.2 | Intensive care unit hospitalization and non-invasive ventilation |
| 8 | 52 | M | Nirmatrelvir/  ritonavir | Severe combined immunodeficiency | 5 | Tixagevimab/cilgavimab | Uninterpretable | BA.2 | Hospitalised and no oxygen therapy |
| 9 | 68 | F | Nirmatrelvir/  ritonavir | Lung adenocarcinoma treated by chemotherapy | 2 | No | 195 | BQ.1.1 | Hospitalised and required low flow oxygen supplementation |
| 10 | 90 | M | Tixagevimab/  cilgavimab | Vexas syndrom treated by rituximab | 3 | No | < 7 | BA.4/5 | Covid-19-related death |
